# Risk factors for atherosclerotic cardiovascular disease (ASCVD) in healthcare professionals of Azar Cohort Study: A cross-sectional Study

**DOI:** 10.34172/hpp.42568

**Published:** 2024-07-29

**Authors:** Neda Roshanravan, Helda Tutunchi, Rezayat Parvizi, Sepideh Bastani, Mohammad Asghari Jafarabadi, Elnaz Faramarzi, Naimeh Mesri Alamdari, Zohreh Ghoreishy, Faezeh Tarighat, Kazem Mehravani, Milad Vahedinezhad, Nazli Namazi, Samad Ghaffari

**Affiliations:** ^1^Drug Applied Research Center, Tabriz University of Medical Sciences, Tabriz, Iran; ^2^Cardiovascular Research Center, Tabriz University of Medical Sciences, Tabriz, Iran; ^3^Endocrine Research Center, Tabriz University of Medical Sciences, Tabriz, Iran; ^4^Stem Cell and Regenerative Medicine Institute, Tabriz University of Medical Sciences, Tabriz, Iran; ^5^Cabrini Research, Cabrini Health, Malvern, VIC, 3144, Australia; ^6^School of Public Health and Preventive Medicine, Monash University, Melbourne, VIC, 3004, Australia; ^7^Department of Psychiatry, School of Clinical Sciences, Monash University, Clayton, VIC, 3168, Australia; ^8^Road Traffic Injury Research Center, Tabriz University of Medical Sciences, Tabriz, Iran; ^9^Liver and Gastrointestinal Diseases Research Center, Tabriz University of Medical Sciences, Tabriz, Iran; ^10^Nutrition Research Center, Tabriz University of Medical Sciences, Tabriz, Iran; ^11^Student Research Committee, Tabriz University of Medical Sciences, Tabriz, Iran; ^12^Rajaie Cardiovascular Medical and Research Center, School of Medicine, Iran University of Medical Sciences, Tehran, Iran; ^13^Diabetes Research Center, Endocrinology and Metabolism Clinical Sciences Institute, Tehran University of Medical Sciences, Tehran, Iran

**Keywords:** Cardiovascular disease, Atherosclerosis, Healthcare

## Abstract

**Background:**

Atherosclerotic cardiovascular disease (ASCVD) is considered a worldwide health problem associated with high morbidity, mortality, and cost of care. In the present study, we examined risk-enhancing factors for ASCVD in healthcare workers of the AZAR cohort population.

**Methods:**

Data from a total of 500 participants were used for this cross-sectional study. Demographic characteristics, anthropometric indices, biochemical factors, and blood pressure were assessed. To evaluate the associations of ASCVD with the parameters mentioned above, univariate and multivariate logistic regression analyses were conducted.

**Results:**

The total frequency of subjects with severe (≥7.5) and low (<7.5) ASCVD was 7.6% (95% CI: 5.4-10.3), and 90.6% (95% CI: 87.7-93.0), respectively. The top strongest links were found between ASCVD and atherogenic index of plasma (AIP) (odds ratio [OR]: 12.8, 95% CI: 3.2-49.9), diabetes (OR: 7.6, 95% CI: 2.8-25), and daily smoking (OR: 7.0, 95% CI: 2.8-20). Based on a multivariate logistic regression model, low-density lipoprotein cholesterol (LDL-C)/apolipoprotein B (Apo b), diabetes, hematocrit, age, Triglycerides (TG)/high-density lipoprotein cholesterol (HDL-C), systolic blood pressure, HDL-C, apolipoprotein A-I (Apo A-I), hemoglobin, and Apo B/Apo A-I have significant associations with ASCVD severity.

**Conclusion:**

In conclusion, the present study showed significant associations between the severity of ASCVD with some parameters among healthcare workers of AZAR cohort study.

## Introduction

 Atherosclerosis, a chronic inflammatory disease of the arteries, is the major cause of cardiovascular diseases (CVDs). Atherosclerotic CVD (ASCVD) is one of the common non-communicable diseases (NCDs) caused by atherosclerotic plaque formation inside the lining of the artery wall and includes coronary heart disease, stroke, and peripheral arterial disease.^1^ In addition, ASCVD is considered a worldwide health problem associated with high morbidity, mortality, and cost of care.^2-4^ Given the dramatic increase in the number of individuals at risk of developing ASCVD throughout the developed and the developing world, identification of risk-enhancing factors for ASCVDis crucial in terms of personal and community health, as some risk factors can be modified, and can prevent or slow down the progression of the disease.^5,6^

 ASCVD includes many common risk factors, such as cigarette smoking, unhealthy diet,sedentary lifestyle, obesity, family history of ASCVD, dyslipidemia, diabetes mellitus, metabolic syndrome (MetS), and hypertension.^7,8^ However, according to recent studies, a substantial number of individuals without these risk factors still develop ASCVD, indicating the importance of identifying unconventional and unknown risk factors for ASCVD in the general population. Identification of these novel risk factors and improving prevention and treatment strategies not only decreases the incidence of ASCVD but also reduces the risk of recurrent cardiovascular events among patients with pre-existing ASCVD.^9-12^

 Although clinical and experimental data have shown association between different biochemical factors such as cholesterol, especially low-density lipoprotein cholesterol (LDL-C), high-density lipoprotein cholesterol (HDL-C), triglyceride (TG), apolipoprotein B (Apo B), and lipoprotein (a) [Lp (a)] concentrations with CVD risk,^13-15^ to the best of our knowledge, few population-based studies have examined the associations of various demographic factors, biochemical parameters, inflammatory biomarkers, as well as lipoprotein ratios or “atherogenic indices” with ASCVD risk. On the other hand, it appears that the patterns of associations in Asian countries may differ from those in Western countries because of differences in lifestyle, socio-economic, and environmental factors. Moreover, a number of multivariate risk models have been developed for ASCVD risk prediction based on Western societies.^8^ For example, the updated 2019 American College of Cardiology (ACC)/American Heart Association (AHA) guideline recommended routinely assessing the 10-year risk of ASCVD by using the pooled cohort equations.^16^In the present study, for the first time, we aimed to examine risk-enhancing factors for ASCVD in healthcare workers of the AZAR cohort study.

## Materials and Methods

 In this cross-sectional study, we obtained data from healthcare professionals of the cohort study, which is part of a large prospective epidemiological research in Iran (the Persian Cohort Study). The cohort study was carried out on healthcare providers, official staff, and university faculty members of Tabriz University of Medical Sciences (TBZMED) as a part of the Azar cohort study, which was conducted by the Liver and Gastrointestinal Diseases Research Center of TBZMED.^15^ Our baseline assessment consisted of a face-to-face health interview and a health examination in terms of a broad range of established and novel risk factors of NCDs. For this cross-sectional investigation, we enrolled around 500 individuals from the Persian cohort investigation between January and June 2019. Written informed consent was obtained from all the participants, and the study was approved by the Ethics Committee of TBZMED (IR.TBZMED.REC.1398.027). Participants of this study include full-time and long-term contract employees aged 18 to 65, who are not pregnant or breastfeeding, and who are not planning to retire within the next five years.

###  Demographic characteristics of the participants

 We assessed the demographic factors—age, gender, marital status, and educational attainment—using a questionnaire. Additionally, the questionnaire investigated lifestyle habits such as alcohol intake, drug and/ or smoking usage, hookah use, and passive smoking.

###  Anthropometric and blood pressure measurements 

 The weight, height, and waist circumference of all the subjects were measured, and their body mass index (BMI) was determined using the standard formula, weight (kg)/height^2^ (m). Using a mercury sphygmomanometer (Rudolf Richter, DE-72417, Germany), a qualified nurse took the participants’ blood pressure twice at a two-minute interval, meaning that the measurements were taken twice for each arm, while the individuals were seated and after ten minutes of rest. The systolic and diastolic blood pressures were determined using the average values, which were then utilized in the study.

###  Biochemical factors

 Blood samples were taken from the participants after an overnight fast ( ≥ 12 hours) to measure the levels of creatinine, blood urea nitrogen (BUN), TG, HDL-C, total cholesterol (TC), serum uric acid (SUA), and fasting blood sugar (FBS) using enzymatic techniques. Additionally, the Friedewald formula (FF) was used to compute the LDL-C values. The triglyceride glucose (TyG) index was calculated using the following formula: ^14,17^

 TyG = Ln [fasting TG (mg/dL) × fasting plasma glucose (mg/dL)/2]. The atherogenic index of plasma (AIP) was estimated according to the corresponding equation as follows:

$$AIP\;=\;Log\;\left(\frac{TG}{HDL\;-\;C}\right)$$

AIP is categorized as low risk: AIP < 0.11; intermediate risk: AIP = 0.11-0.21; high risk: AIP > 0.21.^18^

###  ASCVD risk

 It was calculated based on the ASCVD items and the patients’ 10-year ASCVD risk.^19^ Ten-year risk for ASCVD is categorized as: < 7.5, low; ≥ 7.5 high.

###  Diagnostic criteria for metabolic syndrome

 Based on the National Cholesterol Education Program’s Adult Treatment Panel III report (ATPIII) criteria 2005, determination of Mets was done.^20^ The subjects with three or more of the following criteria were diagnosed as having Mets: increased waist circumference ≥ 102 cm (40 inches) in men and ≥ 88 cm (35 inches) in women, elevated TG ≥ 150 mg/dL (or drug treatment for elevated triglycerides), reduced HDL-C < 40 mg/dL in men and < 50 mg/dL in women; elevated blood pressure ≥ 130/85 mm Hg ( or antihypertensive drug treatment in a patient with a history of hypertension); and elevated FBS ≥ 100 mg/dL (or drug treatment for elevated glucose).

###  Statistical analysis

 All statistical analysis was performed using IBM SPSS Statistics Software (version 24, IBM SPSS Statistics, Armonk, USA). Data were presented as mean (SD) and frequency (percentage) for quantitative and qualitative variables, respectively. ASCVD severity was considered as the dependent outcome of interest. A series of univariate and multivariate logistic regression models were fitted to investigate the relationship between the ASCVD severity and participants’ related variables with this outcome. The univariate and multivariate models were used to compute un-adjusted and adjusted odds ratios (ORs) and their 95% confidence intervals. In the univariate analyses, each variable was entered individually, and in the next step for multivariate analyses, a backward elimination multiple logistic regression was performed to find the set of best predictors of ASCVD severity. *P* values < 0.05 were considered significant.

## Results

 General characteristics of participants are outlined inTable 1.Of 500 subjects, 63.8% were men, and 36.2% were women. The prevalence of MetS in the participants of the study was 22.8%. Four percent of participants suffered from diabetes mellitus and about 8% of them were under treatment by anti-hypertension medications.

 Regarding biochemical parameters, mean serum levels of TC and TG were 178.2 ± 38.7 and 126.3 ± 60.0 mg/dL, respectively (Table 2). Based on the classifications of ACC/AHA for ASCVD, the total frequency of subjects with severe ( ≥ 7.5) and low ( < 7.5) ASCVD was 7.6% and 90.6%, respectively.

 In Table 3, the associations between ASCVD severity and the characteristics of the participants were represented. Apart from the components of ASCVD calculation, several general characteristics and biochemical parameters were linked with this index. As shown in Table 3,the top three strongest links were found between ASCVD and AIP (OR: 12.8, 95% CI: 3.2, 49.9), diabetes (OR: 7.6, 95% CI: 2.8, 25), and daily smoking (OR: 7.0, 95% CI: 2.8, 20). No link was observed between ASCVD severity and FBS (*P* = 0.088), neutrophil to lymphocyte ratio (NLR) (*P* = 0.6), platelet-to-lymphocyte ratio (PLR) (*P* = 0.8), BMI (*P* = 0.5), high-sensitivity C-reactive protein (hs-CRP) (*P* = 0.3), apolipoprotein A1 (ApoA1) (*P* = 0.06), and Apo B (*P* = 0.2).

**Table 1:** General characteristics and biochemical parameters of participants

**Variables**	**Total (n=500)**
Age, years	43.24 ± 7.23
BMI, kg/m^2^	28.08 ± 4.03
Current smoker (daily) %	40 (8.0)
Diabetes mellitus, %	21 (4.2)
Treatment for hypertension, %	38 (7.6)
SBP, mm Hg	115.47 ± 14.46
DBP, mm Hg	75.5 ± 9.49
FBS, mg/dL	87.20 ± 22.33
Hs-CRP, mg/L	2.00 ± 1.59
Insulin, µIU/mL	9.49 ± 8.27
NLR	1.61 ± 0.68
PLR	6.04 ± 2.01
Metabolic syndrome, %	114 (22.8)
TyG index	-0.39 ± 0.41
WC, cm	71 ± 9.37

BMI: body mass index; SBP: systolic blood pressure; DBP: diastolic blood pressure; FBS: fasting blood sugar; Hs-CRP: high sensitivity C-reactive protein; NLR: neutrophil-to-lymphocyte ratio; PLR: platelet-to-lymphocyte ratio; TyG: triglyceride-glucose; WC: waist circumference. Qualitative variables are reported as frequency (percent). Quantitative variables are reported as mean ± SD.

**Table 2:** Baseline lipid parameters of the study population

**Variables**	**Total (n=500)**
Total cholesterol, mmol/L	178.26 ± 38.71
LDL-C, mmol/L	107.79 ± 31.57
HDL-C, mmol/L	45.19 ± 10.86
LDL-C/HDL-C	2.49 ± 0.94
Non-HDL-C, mg/dL	133.07 ± 35.64
Non-HDL-C/HDL-C	3.10 ± 1.20
TG, mg/dL	126.38 ± 60.02
ApoA1	117.63 ± 20.72
ApoB	86.03 ± 12.28
LDL-C/ApoB	1.27 ± 0.42
Apo B/ApoA1	0.76 ± 0.32
AIP	0.42 ± 0.23
Statins use, n (%)	11 (2.2%)

LDL-C: low-density lipoprotein cholesterol; HDL-C: high-density lipoprotein cholesterol; TG, triglyceride; APO B: apolipoprotein B; ApoA1: apolipoprotein A1; AIP: atherogenic index of plasma. Data are reported as mean ± SD.

**Table 3:** Association between ASCVD severity and participants’ characteristics

**Variables**	**Univariate**		**Multivariate ** ^*^	
	**Odds Ratio (95% CI)**	***P*** ** value**	**Odds Ratio (95% CI)**	***P*** ** value**
Age, years	1.35 (1.25-1.46)	< 0.001	2.03 (1.49-2.75)	< 0.001
Total cholesterol, mmol/L	1.01 (1.00-1.02)	0.005	-	-
HDL-C, mmol/L	0.95 (0.92-0.98)	0.010	0.84 (0.75-0.94)	0.016
LDL-C, mmol/L	1.01 (1.00-1.02)	0.002	-	-
Daily smoking	7.04 (2.85-20)	< 0.001	-	-
Hypertension	2.54 (1.09-5.89)	0.030	-	-
Diabetes	7.63 (2.85-25)	< 0.001	12.05 (1.81-80.07)	0.031
FBS, mg/dL	1.00 (0.99-1.01)	0.088	-	-
TG, mg/dL	1.00 (1.00-1.01)	0.001	-	-
TG/HDL-C	1.22 (1.08-1.37)	0.001	1.79 (1.25-2.56)	0.007
Total cholesterol/HDL-C	1.59 (1.16-2.19)	0.004	-	-
LDL-C/HDL-C	1.67 (1.12-2.49)	0.012	-	
Non-HDL-C	1.01 (1.00-1.02)	< 0.001	-	-
Non-HDL-C/HDL-C	1.59 (1.16-2.19)	0.004	-	-
LDL-C/Apo B	2.24 (1.09-4.61)	0.027	-	-
AIP	12.80 (3.28-49.90)	< 0.001	-	-
RBC	2.75 (1.53-4.97)	0.001	-	-
Hemoglobin	1.27 (1.03-1.57)	0.024	0.08 (0.012-0.587)	0.036
Hematocrit	1.14 (1.05-1.24)	0.001	2.36 (1.18-4.71)	0.040
NLR	1.11 (0.71-1.73)	0.638	-	-
PLR	0.08 (0.00-0.89)	0.896	-	-
BMI	1.12 (0.74-1.69)	0.591	-	-
Hs-CRP	1.07(0.90-1.28)	0.399	-	-
ApoA1	0.98 (0.97-1.00)	0.062	0.63 (0.48-0.83)	0.006
ApoB	1.01 (0.988-1.041)	0.288	1.85 (1.31-2.61)	0.003
MetS	3.34 (1.70-6.57)	< 0.001	-	-
SBP	1.06 (1.04-1.09)	< 0.001	-	-
DBP	1.07 (1.04-1.11)	< 0.001	-	-
TyG index	2.28(1.07-4.85)	0.032	-	-
WC	1.02(0.98-1.05)	0.252	-	-
Blood urea	1.16 (1.06-1.27)	0.001	**-**	**-**
Creatinine	10.60(1.42-79.03)	0.021	**-**	**-**

ASCVD: Atherosclerotic cardiovascular disease; HDL-C: high-density lipoprotein cholesterol; LDL-C: low-density lipoprotein cholesterol; FBS: fasting blood sugar; TG: triglyceride; APO B: apolipoprotein B; ApoA1: apolipoprotein A1; AIP: atherogenic index of plasma; RBC: red blood cells; NLR: neutrophil-to-lymphocyte ratio; PLR: platelet-to-lymphocyte ratio, BMI: body mass index; Hs-CRP: high sensitivity C-reactive protein; MetS: metabolic syndrome; SBP: systolic blood pressure; DBP: diastolic blood pressure, TyG: triglyceride-glucose; WC: waist circumference; CI: confidence interval; L: lower bound; U: upper bound. Dependent Variable: ASCVD severity. < 7.5 low; ≥ 7.5 high. Due to collinearity, LDL-C/ Apo B and ApoB/ ApoA1 were removed from the model. * Variables remained in the model in the final step of the logistic model with a backward strategy.

 Based on the logistic model with backward strategy finally, 9 variables remained in the model. Findings show that diabetes (OR: 12.0, 95% CI: 1.81, 80.0) has the strongest associations with ASCVD severity.

## Discussion

 The present cross-sectional study showed that apart from the components of ASCVD, significant associations were found between the severity of ASCVD and TG/HDL-C, ApoA1, ApoB, hemoglobin, and hematocrit in Iranian healthcare workers.

 To the best of our knowledge, few studies have examined the associations of various biochemical parameters with ASCVD, particularly in population-based studies. Identifying factors linked with an increased risk of ASCVD is critical for developing effective interventions.^21^ A number of studies have examined the effects of various cardiovascular risk factors, and some introduced specific indices that consist of several parameters. The Framingham risk score, for instance, was developed by the Framingham Heart Study to estimate the 10-year risk of coronary artery disease using seven items (age, sex, TC, HDL-C, diabetes mellitus, blood pressure, and smoking). Another prediction model to estimate the risk of CVDs and to classify high-risk groups is ASCVD. This index includes 9 parameters such as age, race, sex, systolic blood pressure, TC, HDL-C, smoking, and any medical therapy for hypertension or diabetes mellitus. However, such predictive models have been developed based on Western societies, and might not be practical in Asian countries. In addition, some additional biochemical or demographic characteristics may be associated with an increased risk of ASCVD.

 Accordingly, exploring the association between various factors with these predictive models can clarify the roles of new parameters in the risk of ASCVD; and it may lead to developing a new model based on the characteristics of societies. For instance, Choi, showed that for primary prevention, adults aged 40 to 75 with LDL-C concentrations between 70 and 189 mg/dL, ankle-brachial index < 0.9, high hs-CRP ( ≥ 2.0 mg/L), apoB ≥ 130 mg/dL (with persistent hyper-triglycerides), and Lp (a) ≥ 50 mg/dL can predict the risk of ASCVD in Korean society. They recommended statin therapy in the borderline risk group.^21^ Regarding the direct link between ASCVD and Apo-B, our study was in line with the study by Choi.^21^

 Jialal and Chaudhuri suggested targeting inflammatory parameters such as hs-CRP at reducing ASCVD in patients with type 2 diabetes.^22^ However, our findings were inconsistent and no link was found between hs-CRP and ASCVD. These conflicting findings may be due to differences in the target population. In Jialal and Chaudhuri study, only patients with type 2 diabetes were included. According to a cohort research in the Chinese population, diabetic patients with HbA1c levels between 7.0 and 8.0% had a greater CVD risk, even if their ASCVD was moderate.^23^ In the study by Rossello et al, it was revealed that routine measurement of HbA1c can identify asymptomatic subjects who have a higher risk for subclinical atherosclerosis on top of traditional cardiovascular risk factors.^24^ Khan et al also concluded that there is a link between high levels of HbA1c and the degree of coronary artery disease in patients with diabetes. However, as it was a cross-sectional study, the cause-and-effect link cannot be confirmed.^25^ In a recent retrospective study in the Chinese population, it was found that various levels of HbA1c are likely to be indicators for carotid artery plaques and thus, it should be taken into account in patients with coronary heart disease.^26^ Similar findings were also reported in another study in Chinese population.^27^ However, in our study, participants were included, no matter if they were suffering from diabetes, and no measurement for HbA1C was conducted. The TyG index, a parameter derived from the FBS and TG concentration, has been proposed as a convincing indicator of insulin resistance recently. Based on observational studies, the TyG index was positively associated with ASCVD risk in the general population.^15^ In this investigation, we discovered a substantial link between TyG index and ASCVD severity, although the link dissipated in a multivariate logistic model. Regarding the association between the serum levels of TG and ASCVD, an overview revealed that there is a positive link. Considering previous findings, lifestyle modification and statin therapy are recommended to modulate this parameter.^28^ Although in the present study a statistically significant association was found between serum levels of TG and ASCVD severity, this significance did not remain in a multivariate logistic model. Furthermore, we obtained a positive link between ASCVD severity and TG/HDL-C ratio. In addition, we obtained positive links between ASCVD, hemoglobin, and hematocrit. In a retrospective cohort study on the NHIS‐HEALS (National Health Insurance Service–National Health Screening) Cohort study on men (n = 170 078) and women (n = 122 116) aged above 40 without CVD, a positive link between low or high hemoglobin concentrations and cardiovascular and all-cause mortality was found. However, maintaining hemoglobin concentrations within normal range was associated with decreased all-cause mortality.^29^

 In this realm, one possible mechanism is the effects of ventricular remodeling and cardiac dysfunction following anemic status. In addition, anemia is likely to be a marker for an inflammatory process which can result in a high risk of CVD events. On the other hand, the viscosity of blood is primarily exerted by red blood cells. Greater hematocrit concentrations can thicken the blood and slow down the flow rate, increase the peripheral resistance, and decrease blood flow and perfusion to different tissues.^29^

 Generally, healthcare providers should incorporate suitable screening tools to identify individuals at higher risk of ASCVD with the measurement of anthropometric indices and biochemical parameters. For individuals at high metabolic risk, 10-year ASCVD risk should be checked to decide on therapy for the reduction of lipoproteins, lipid profile, and hypertension. Subjects with prediabetes should be checked, at least annually, for diabetes mellitus, and referred to dietitians to receive an individualized diet and physical activity program. Lifestyle management is the priority to reduce ASCVD. Overweight and obese subjects should lose at least 5% of initial body weight. Apart from these strategies, behavior adjustments, as well as pharmacological and medical therapy, might be used when lifestyle changes alone are insufficient to accomplish the required goals.^7,30^

 The present study has two main limitations that should be addressed. First, the 10-year ASCVD risk score was developed based on Western populations, and the identification of additional risk factors using the examination of any associations with ASCVD might lead to inappropriate predictors for our society. Second, the type of medications might have affected the associations; but they were not reported. In addition, a relatively large sample size was examined to increase the generalization of the findings to our society.

## Conclusion

 The present cross-sectional study revealed that age, several biochemical parameters including HDL-C, hemoglobin, hematocrit, Apo A-I, Apo B, and some biochemical indices (TG/HDL-C,) are associated with the severity of ASCVD. In addition, there is a strong link between diabetes and the severity of ASCVD in healthcare workers in East Azerbaijan Iran. Due to the effects of age, lifestyle, race, and genetics on the risk-enhancing factors for ASCVD, further studies in other societies are suggested.

## Acknowledgments

 The authors are thankful to the Cardiovascular Research Center and Liver and Gastrointestinal Diseases Research Center Tabriz University of Medical Sciences for permission to conduct this research. The authors also would like to appreciate the cooperation of clinical research development unit, Shahid Madani Hospital, Tabriz, Iran in conducting this research.

## Competing Interests

 The authors declare no conflicts of interest.

## Ethical Approval

 The study was approved by the ethics committee of Tabriz University of Medical Sciences (IR.TBZMED.REC.1398.027).
